# Simple sequence repeats in *Helicobacter canadensis *and their role in phase variable expression and *C*-terminal sequence switching

**DOI:** 10.1186/1471-2164-11-67

**Published:** 2010-01-27

**Authors:** Lori AS Snyder, Nicholas J Loman, James D Linton, Rebecca R Langdon, George M Weinstock, Brendan W Wren, Mark J Pallen

**Affiliations:** 1 Centre for Systems Biology, School of Biosciences, University of Birmingham, Edgbaston, Birmingham, B15 2TT, UK; 2 School of Life Sciences, Kingston University, Kingston upon Thames, KT1 2EE, UK; 3 Faculty of Life Sciences, University of Manchester, Michael Smith Building, Oxford Road, Manchester, M13 9PT, UK; 4 Pathogen Molecular Biology Unit, London School of Hygiene and Tropical Medicine, Keppel Street, London, WC1E 7HT, UK; 5 The Genome Center, Washington University School of Medicine, St. Louis, Missouri, USA

## Abstract

**Background:**

*Helicobacter canadensis *is an emerging human pathogen and zoonotic agent. The genome of *H. canadensis *was sequenced previously and determined to contain 29 annotated coding regions associated with homopolymeric tracts.

**Results:**

Twenty-one of the repeat-associated coding regions were determined to be potentially transcriptionally or translationally phase variable. In each case the homopolymeric tract was within the predicted promoter region or at the 5' end of the coding region, respectively. However, eight coding sequences were identified with simple sequence repeats toward the 3' end of the open reading frame. In these cases, the repeat tract would be too far into the coding region to be mediating translational phase variation. All of the 29 coding region-associated homopolymeric tracts display variability in tract length in the sequencing read data.

**Conclusions:**

Twenty-nine coding regions have been identified in the genome sequence of *Helicobacter canadensis *strain NCTC13241 that show variations in homopolymeric tract length in the bacterial population, indicative of phase variation. Five of these are potentially associated with promoter regions, which would lead to transcriptional phase variation. Translational phase variation usually switches expression of a gene ON and OFF due to the repeat region being located sufficiently close to the initiation codon for the resulting frame-shift to lead to a premature termination codon and stop the translation of the protein. Sixteen of the 29 coding regions have homopolymeric tracts characteristic of translational phase variation. For eight coding sequences with repeats located later in the reading frame, changes in the repeat tract length would alter the protein sequence at the *C*-terminus but not stop the expression of the protein. This mechanism of *C*-terminal phase variation has implications for stochastic switching of protein sequence in bacterial species that already undergo transcriptional and translational phase variation.

## Background

The gastric pathogen *Helicobacter pylori *was the first identified *Helicobacter *species, was first cultured in 1984, and is associated with peptic ulcers, mucosa-associated lymphoid tissue lymphomas, chronic active gastritis, chronic atrophic gastritis and subsequent carcinomas, persistent diarrhoea, and increased susceptibility to other diseases [1-3]. Since 1984, over 20 further *Helicobacter *species have been identified, many of which have been implicated as animal pathogens, but in general these remain poorly characterised.

To date, three *H. pylori *genome sequences have been published, one each isolated from cases of gastritis [4], duodenal ulcer [5], and chronic atrophic gastritis [6]. Five additional genome sequences, not yet completed and published, are from three cases of gastric carcinoma, one from gastric ulcer, and one from a remote Amazonian village. But humans are not the only species plagued by gut problems associated with these bacteria; *Helicobacter acinonychis*, the genome sequence of which was published in 2006 [7], is believed to contribute to the severe gastritis experienced by cheetahs and other big cats that can lead to premature death of these felines in captivity [8,9]. At the other end of the mammalian spectrum, mice colonies with increased incidence of liver tumours were found to be colonized with *Helicobacter hepaticus *[10,11], for which strain ATCC 51449 has been genome sequenced [11]. In addition, the *Helicobacteraceae *includes *Wolinella succinogenes*, a non-pathogen that can be isolated from the rumen of cattle [12] and considered to be phylogenetically an intermediate between *Helicobacter *and *Campylobacter *[13]. A genome sequence is available for *W. succinogenes *strain DSM1740 [14].

Some isolates which might have previously been taken for known *Helicobacter *spp. or *Campylobacter *spp. are instead novel *Helicobacter *species [15]. *Helicobacter pullorum *has been repeatedly isolated from chickens and detected in chicken faeces by molecular methods [16-21]. This species is also found in humans [18,20,22-25], where it is associated with gastroenteritis, liver disease, diarrhoea, and gall bladder disease [20,24-26]. *H. pullorum *differs from most other Helicobacter spp. in that it lacks a flagellar sheath and is unable to hydrolyze indoxyl acetate [20]. It is this later phenotype that eventually revealed that some of the isolates being classified as *H. pullorum *were actually a novel species, *Helicobacter canadensis *[18,20,24] which is indoxyl acetate positive [24]. Labelled an emerging pathogen as a zoonotic agent [27], *H. canadensis *has been isolated from geese [27], rodent faeces [28], swine [29], and humans, where it has been associated with diarrhoea and bacteraemia [30,31].

During the annotation of the complete genome sequence of *H. canadensis *strain NCTC13241 [32], simple sequence repeats were sought and a repertoire of 29 homopolymeric tract-associated coding regions was identified. Five candidates for transcriptional phase variation and 16 candidates for translational phase variation were identified. The remaining eight annotated coding sequences were identified with long poly-G tracts (≥ 10 bp) toward the end of the annotated coding region. These represent a novel mechanism for phase variation in which stochastic switching of simple sequence repeat tract lengths mediates changes in expressed protein sequence. This is distinct from transcriptional phase variation, in which the distance and facing of promoter elements are altered, and from translational phase variation, in which frame-shifts resulting from repeat tract length changes lead to premature termination. We propose that this *C*-terminal phase variation may also be found in other species employing transcriptional and translational phase variation.

## Results and Discussion

### Search for phase variable genes

Given the presence of an extensive repertoire of phase variable genes in other *Helicobacter *spp. [4,5,11,33,34], simple sequence repeats were sought in the *H. canadensis *strain NCTC13241 genome sequence data. If repeats were discovered to be within the context of a predicted coding region or predicted promoter such that they had the potential to mediate phase variation, then that feature was annotated as potentially phase variable (Table 1).

**Table 1:** Phase variable gene candidates in *H. canadensis *strain NCTC 13241.

Tract *	Range†	Candidacy ‡	Location	Locus	Description
(G)10	(G)8 - 11	strong	within gene	HCAN_0054	hypothetical protein, frame-shifted
(G)14	(G)11 - 17	possible promoter	promoter	HCAN_0075	hypothetical
(G)11	(G)9 - 14	good	within gene	HCAN_0101	glycosyl transferase
(G)9	(G)8 - 10	strong	within gene	HCAN_0115	acetyl-coenzyme A synthetase 2, frame-shifted
(G)13	(G)11 - 15	strong	within gene	HCAN_0151	hypothetical protein, frame-shifted
(G)12	(G)9 - 14	strong	within gene	HCAN_0153	polysaccharide deacetylase family protein, frame-shifted
(A)12	(A)10 - 14	possible promoter	promoter	HCAN_0162	conserved hypothetical
(G)14	(G)12 - 16	possible promoter	promoter	HCAN_0234	immunoglobulin A1 protease precursor
(G)14	(G)11 - 16	putative	within gene ¶	HCAN_0344	putative filamentous haemagglutinin domain protein
(G)14	(G)10 - 16	good	within gene	HCAN_0346	conserved hypothetical protein
(G)16	(G)12 - 20	possible promoter	promoter	HCAN_0457	vacuolating cytotoxin precursor
(G)14	(G)11 - 16	good	within gene	HCAN_0632	putative oxidoreductase
(G)11	(G)9 - 12	good	within gene	HCAN_0647	putative methyltransferase
(G)12	(G)10 - 14	strong	within gene	HCAN_0648	conserved hypothetical protein, frame-shifted
(G)11	(G)10 - 12	good	within gene	HCAN_0651	conserved hypothetical protein
(G)11	(G)9 - 13	good	within gene	HCAN_0655	hypothetical protein
(G)9	(G)7 - 11	putative	within gene	HCAN_0659	2-polyprenyl-3-methyl-5-hydroxy-6-metoxy-1,4-benzoquinol methylase
(G)11	(G)9 - 12	good	within gene	HCAN_0670	hypothetical protein
(G)13	(G)11 - 14	strong	within gene	HCAN_0671	methyltransferase, frame-shifted
(G)13	(G)10 - 14	possible promoter	promoter	HCAN_0714	vacuolating cytotoxin precursor
(G)13	(G)10 - 14	good	within gene	HCAN_1335	conserved hypothetical protein

* Tract length from accession number CM000776 [32].

† Range is the range of repeat copy number observed in the sequence read data (see Additional Files files 1, 2, 3, 4, 5, 6, 7, 8, 9, 10, 11, 12, 13, 14, 15, 16, 17, 18, 19, 20 and 21).

‡ Candidacy is based on the presence of a frame-shift and the length of the repeat tract.

¶This would be a strong candidate for phase variation if an alternative initiation codon 5' of the repeat was chosen rather than that annotated.

Based on previous investigations [33-35], searches were made of the genome sequence data for homopolymeric tracts greater than or equal to (G)7, (C)7, (A)9, and (T)9 and for dinucleotide repeat tracts with five or more copies of the dinucleotide. No potential dinucleotide-mediated phase variable CDSs were identified in the *H. canadensis *genome sequence data. For all homopolymeric tracts the context of the repeat, tract length, and presence of a frame-shift were assessed to determine that the *H. canadensis *genome sequence contains 21 potential phase variable genes (Table 1). Of these, six are strong candidates, eight are good candidates, and two are putative candidates for translational phase variation, while five are possibly promoter-associated, mediating transcriptional phase variation. Strong candidates contained frame-shift mutations or long tract lengths (>9), or both. Good candidates contained long tracts in the appropriate position to mediate translational switching. One of the putative candidates (HCAN_0659) contained a shorter (G)9 tract length and no frame-shift, while the other (HCAN_0344) would be considered a strong candidate if an alternative initiation codon 5' of the repeat were chosen rather than that annotated.

It is interesting to note that in all cases the translational phase variation is mediated by poly-G tracts, whereas candidates with poly-C, -A, -T, -GA, -CT, -TC, -AT, and -AG tracts have been reported in other *Helicobacter *spp. [4,5,11,33,34]. The sole occurrence of a poly-A tract potentially involved in phase variation in this *H. canadensis *is in the putative promoter region of HCAN_0162, a conserved hypothetical gene.

### *C*-terminal phase variation

In addition to the potentially phase variable genes identified, eight CDSs were found to contain homopolymeric tracts at the 3' end (Table 2) of the reading frame. In this location these could not be mediating phase variable expression of the CDS as most of the mRNA would be translated before reaching the late homopolymeric tract and subsequent termination codon. In most cases, an alteration in the reading frame at that point would change the length of the encoded protein by only a few amino acids (Table 2).

**Table 2:** *C*-terminal phase variable gene candidates in *H. canadensis *strain NCTC 13241.

Tract *	Range†	Frame 1 ‡	Frame 2 ‡	Frame 3 ‡	CDS length in amino acids	Locus TagG	ene	Description
(G)14	(G)11 - 17	4	1	**18**	322	HCAN_0165	rfbB	NAD-dependent epimerase/dehydratase
(G)13	(G)10 - 16	17	4	**2**	267	HCAN_0641		3-oxoacyl-[acyl-carrier-protein] reductase
(G)11	(G)8 - 14	17	18	**22**	317	HCAN_0643		Methionyl-tRNA Formyl-transferase
(G)12	(G)10 - 14	5	1	**50**	429	HCAN_0653		hypothetical protein
(G)13	(G)11 - 16	**14**	104	25	382	HCAN_0660		hypothetical protein
(G)10	(G)8 - 12	1	**35**	2	243	HCAN_0665		conserved hypothetical protein
(G)11	(G)9 - 13	**2**	0	16	155	HCAN_0914	flaG	flagellar proteinG
(G)12	(G)10 - 13	**17**	19	30	445	HCAN_1332		conserved

* Tract length from accession number CM000776 [32].

† Range is the range of repeat copy number observed in the sequence read data (see Additional Files 22, 23, 24, 25, 26, 27, 28 and 29).

‡ Frame is the number of amino acids after the homopolymeric tract before the termination codon; bold is the annotated *C*-terminus.

It is interesting to note that, like the transcriptional and translational phase variable CDSs, all of these *C- *terminal variation CDSs contain poly-G tracts and all are (G)10 or more (Table 2). Given the strength in poly-G tracts in the transcriptionally and translationally phase variable genes (Table 1) and the length of these tracts (Table 2), this data supports a role for the instability of these tracts at the end of the gene, as well as at the beginning and within the promoter.

One of these, *flaG *(HCAN_0914) contains a (G)11 tract five bases before the termination codon and alteration of the tract length would lead to slight changes in the length of the encoded protein. As annotated, there are two amino acids before the termination codon. In the other two frames there would be 16 amino acids or no amino acids before the termination codons in those frames (Table 2). FlaG has been shown to affect flagellar length and adherence to Hep-2 cells in *Aeromonas *[36]. Thus it is intriguing that changes in the length of this homopolymeric tract would alter the *C*-terminus of this protein.

For HCAN_0660, a change in reading frame could result in a merger of this CDS and the next downstream CDS, HCAN_0661 encoding 82 amino acids. Although both CDSs are currently hypothetical, such an arrangement suggests that the protein sequences may have roles that can alternatively be filled as individual proteins or jointly as one protein. This finding, that a phase variation event can determine whether two proteins are made separately or are translated as one protein, is perhaps unprecedented. It may be that degeneration events have disrupted similar fusions in this or other bacterial species, which are not now apparent from sequence data.

### Population-level variation within homopolymeric tracts

Previous studies into phase variable genes of the *Helicobacter *spp. have indicated that phase variable switching of a gene can be demonstrated at the population level through DNA sequencing. It has been shown that variations in the lengths of homopolymeric tracts are attributable to variations in the DNA template population and are not an artefact of the sequencing process, although this may be influenced by the length of repeat and the experimental conditions [33].

When the read data from the genome sequencing was analyzed for variations in homopolymeric tract length, it was found that all of the repeats investigated showed some degree of variation (Tables 1 &2). While it is known that homopolymeric tracts can be problematic for this sequencing technology (454, Roche), the results are consistent with the level of variation that would be expected at the population level. Phase variable bacteria are known to have dynamically changing populations undergoing phase variation events that lead to intra-population diversity and, in some cases, visible differences on culture plates, including colony sectoring [37]. Variation in repeat lengths at the sequence level should therefore be expected. In each case the range of the repeat lengths observed here is indicated in Tables 1 &2 and the alignments of the read data is available in Additional files 1, 2, 3, 4, 5, 6, 7, 8, 9, 10, 11, 12, 13, 14, 15, 16, 17, 18, 19, 20, 21, 22, 23, 24, 25, 26, 27, 28 and 29).

This level of variation is suggestive evidence of phase variation of these coding regions, indicating that each of these coding regions warrants further study. This evidence is particularly important for those that are putative candidates (Table 1) and those that contain *C*-terminal repeats (Table 2). In all of the translational phase variation candidates the repeat lengths would correspond to both ON and OFF states of full-length gene expression. The highest degree of variability is seen in HCAN_0457, encoding the vacuolating cytotoxin precursor, with 12 to 20 copies of the poly-G repeat.

### Potential structural consequences of *C*-terminal phase variation

Each of the eight CDSs identified as containing a homopolymeric tract at the *C*-terminal end of the reading frame were translated and compared against the NCBI Conserved Domain Database (CDD). In each case where structural data was available, the Cn3D model was assessed to determine what the potential structural consequences would be of alterations in the length and sequence of the *C*-terminus.

The *C*-terminal structures available for proteins which share similarity with HCAN_0165 (*rfbB*) all have an α-helix at the *C*-terminus. This is potentially eliminated in the shorter forms of this CDS in frames 1 and 2, mediated by a poly-G tract.

The structures of proteins with similar conserved domains to HCAN_0641 and HCAN_0914 suggest that changes in the length of this CDS would alter the *C*-terminal β-sheet. HCAN_0914 is *flaG*, therefore this potential change in β-sheet structure may have an effect on the *H. canadensis *flagella.

Changes in the poly-G repeat within HCAN_0643 would not alter the length of this protein, however changes would alter the sequence. In this case, the structure of CDD similar protein Fmt suggests that this would be within a largely unstructured *C*-terminus.

For the remaining four CDSs, there were either no CDD hits (HCAN_0653) or none that were full-length (HCAN_0660, HCAN_0665, and HCAN_1332).

### Distribution of phase variable genes

When the *H. canadensis *strain NCTC13241 genome sequence was investigated to identify novel CDSs not found in other *Helicobacter *genome sequences, only one example of a contiguous cluster longer than 5 genes was found (HCAN_0630 to HCAN_0663). This region is notable for possessing three copies of *asnB*, encoding asparagine synthetase (HCAN_0654, HCAN_0657, and HCAN_0662). A fourth copy of *asnB *(HCAN_0730) is 69 kb outside this cluster adjacent to one of the two STT3 domain-containing PglB copies (HCAN_0729 and HCAN_0930) [32]. Most of the other coding sequences in the region are of unknown function, lacking significant matches in GenBank. Notably, a high frequency of potentially phase variation-mediating homopolymeric tracts were detected in and around this region, with eight out of 21 identified candidate phase variable genes (Table 1) and five out of eight *C*-terminal variable genes (Table 2) being within or near this cluster. These encode a putative oxidoreductase (HCAN_0632), a 3-oxoacyl-[acyl-carrier-protein] reductase (HCAN_0641), a methionyl-tRNA formyltransferase (HCAN_0643), two putatitive methyltransferases (HCAN_0647 and HCAN_0671), a 2-polyprenyl-3-methyl-5-hydroxy-6-metoxy-1,4- benzoquinol methylase (HCAN_0659), three conserved hypothetical proteins (HCAN_0648, HCAN_0651, and HCAN0665), and four hypothetical proteins (HCAN_0653, HCAN_0655, HCAN0660, and HCAN_0670). This cluster of repeat tracts within the CDSs may suggest that this region of the chromosome is a particular hot-spot for the presence of phase variable tracts. This may have functional consequences for the interaction of these gene products or their regulatory controls.

The genome of *H. canadensis *NCTC13241 contains a capsular polysaccharide export locus (HCAN_0144 to HCAN_0149, HCAN_0150) encoding orthologues of KpsS, KpsD, KpsE, KpsT, KpsM, and KpsC from *C. jejuni*. This is the first evidence of a polysaccharide capsule in the *Helicobacter *spp. and, like in *C. jejuni*, this was only revealed once the genome had been sequenced. The presence of an annotated sialyltransferase just outside of this locus (HCAN_0152, *siaD*) suggests capsule sialylation, which is known to be an important virulence factor in *Neisseria meningitidis *[38] and group B *Streptococcus *[39]. In *C. jejuni*, however, only LOS has been shown to be sialylated [40]. Two strong, frame-shifted candidates for translational phase variation (HCAN_0151 and HCAN_0153) flank this sialyltransferase CDS, encoding a hypothetical protein and a polysaccharide deacetylase family protein, respectively.

## Conclusion

*H. canadensis *strain NCTC13241 contains five candidate transcriptional phase variable CDSs, 16 candidate translational phase variable CDSs, and eight candidate *C*-terminal phase variable CDSs. In all cases the read data is indicative of repeat tract length variation in the bacterial population pool collected for DNA extraction and sequencing. A previous study of bacterial genome sequences has suggested that due to their instability, repeat tracts are selected against in coding sequences. When they are present, there is a demonstrated bias toward homopolymeric tracts within the first one-fifth of the coding region [41], the location expected for translational phase variation. This data also shows that there are a higher proportion of homopolymeric tracts in the final one-fifth of the coding region than in the internal three-fifths, which may support *C*-terminal variation in other bacterial species [41]. Alternations of the tract length in the candidate *C*-terminal phase variable *H. canadensis *CDSs would result in differences in the *C*-terminus of the encoded proteins, which may impact the function, specificity, and/or antigenicity of the products. Similarly placed repeats have been identified in the *Neisseria *spp. (Snyder, previously unreported personal observation), which extensively utilizes phase variation for transcriptional and translational expression switching. Indeed, the potential for 3' repeats to generate gene fusions, as seen here between HCAN_0660 and HCAN_0661, has been previously speculated [41]. Such genes containing stochastic switches late in the coding region warrant further investigation in the laboratory. In addition, the genome sequence data from other species that employ phase variation should be re-investigated in light of this finding in *H. canadensis*.

## Methods

Using the DNA sequence search facility within the GenDB annotation software [42], simple sequence repeats greater than or equal to (G)7, (C)7, (A)9, and (T)9 were identified. In addition, dinucleotide repeats greater than or equal to four copies were found. Tandem repeats in the genome sequence data were identified using Tandem Repeats Finder [43]. The context of identified repeats was investigated within GenDB and associated CDSs were annotated as described previously [32]. The NCBI BLASTP search was used to access the Conserved Domain Database and associated links to Cn3D structural data files, which were visualized using Cn3D version 4.1 available from the NCBI.

## Abbreviations

CDD: the NCBI Conserved Domain Database.

## Authors' contributions

All authors contributed to the original genome sequencing project [32] and have read and approved the final manuscript. LS and NL conducted the homopolymeric tract searches. LS analyzed the repeats in their context and wrote the manuscript.

## Supplementary Material

Additional file 1 **HCAN_0054**. Alignment of the sequencing read data associated with the homopolymeric tract within HCAN_0054. Click here for file

Additional file 2 **HCAN_0075**. Alignment of the sequencing read data associated with the homopolymeric tract within HCAN_0075. Click here for file

Additional file 3 **HCAN_0101**. Alignment of the sequencing read data associated with the homopolymeric tract within HCAN_0101. Click here for file

Additional file 4 **HCAN_0115**. Alignment of the sequencing read data associated with the homopolymeric tract within HCAN_0115. Click here for file

Additional file 5 **HCAN_0151**. Alignment of the sequencing read data associated with the homopolymeric tract within HCAN_0151. Click here for file

Additional file 6 **HCAN_0153**. Alignment of the sequencing read data associated with the homopolymeric tract within HCAN_0153. Click here for file

Additional file 7 **HCAN_0162**. Alignment of the sequencing read data associated with the homopolymeric tract within HCAN_0162. Click here for file

Additional file 8 **HCAN_0234**. Alignment of the sequencing read data associated with the homopolymeric tract within HCAN_0234. Click here for file

Additional file 9 **HCAN_0344**. Alignment of the sequencing read data associated with the homopolymeric tract within HCAN_0344. Click here for file

Additional file 10 **HCAN_0346**. Alignment of the sequencing read data associated with the homopolymeric tract within HCAN_0346. Click here for file

Additional file 11 **HCAN_0457**. Alignment of the sequencing read data associated with the homopolymeric tract within HCAN_0457. Click here for file

Additional file 12 **HCAN_0632**. Alignment of the sequencing read data associated with the homopolymeric tract within HCAN_0632. Click here for file

Additional file 13 **HCAN_0647**. Alignment of the sequencing read data associated with the homopolymeric tract within HCAN_0647. Click here for file

Additional file 14 **HCAN_0648**. Alignment of the sequencing read data associated with the homopolymeric tract within HCAN_0648. Click here for file

Additional file 15 **HCAN_0651**. Alignment of the sequencing read data associated with the homopolymeric tract within HCAN_0651. Click here for file

Additional file 16 **HCAN_0655**. Alignment of the sequencing read data associated with the homopolymeric tract within HCAN_0655. Click here for file

Additional file 17 **HCAN_0659**. Alignment of the sequencing read data associated with the homopolymeric tract within HCAN_0659. Click here for file

Additional file 18 **HCAN_0670**. Alignment of the sequencing read data associated with the homopolymeric tract within HCAN_0670. Click here for file

Additional file 19 **HCAN_0671**. Alignment of the sequencing read data associated with the homopolymeric tract within HCAN_0671. Click here for file

Additional file 20 **HCAN_0714**. Alignment of the sequencing read data associated with the homopolymeric tract within HCAN_0714. Click here for file

Additional file 21 **HCAN_1335**. Alignment of the sequencing read data associated with the homopolymeric tract within HCAN_1335. Click here for file

Additional file 22 **HCAN_0165**. Alignment of the sequencing read data associated with the homopolymeric tract within HCAN_0165. Click here for file

Additional file 23 **HCAN_0641**. Alignment of the sequencing read data associated with the homopolymeric tract within HCAN_0641. Click here for file

Additional file 24 **HCAN_0643**. Alignment of the sequencing read data associated with the homopolymeric tract within HCAN_0643. Click here for file

Additional file 25 **HCAN_0653**. Alignment of the sequencing read data associated with the homopolymeric tract within HCAN_0653. Click here for file

Additional file 26 **HCAN_0660**. Alignment of the sequencing read data associated with the homopolymeric tract within HCAN_0660. Click here for file

Additional file 27 **HCAN_0665**. Alignment of the sequencing read data associated with the homopolymeric tract within HCAN_0665. Click here for file

Additional file 28 **HCAN_0914**. Alignment of the sequencing read data associated with the homopolymeric tract within HCAN_0914. Click here for file

Additional file 29 **HCAN_1332**. Alignment of the sequencing read data associated with the homopolymeric tract within HCAN_1332. Click here for file
